# Effect of cardiovascular biofeedback on nursing staff stress: a randomized controlled clinical trial

**DOI:** 10.1590/0034-7167-2023-0069

**Published:** 2023-12-04

**Authors:** Andréia Barcellos Teixeira Macedo, Edwing Alberto Urrea Vega, Liliana Antoniolli, Jessica Morgana Gediel Pinheiro, Juliana Petri Tavares, Sônia Beatriz Cócaro de Souza

**Affiliations:** I Universidade Federal do Rio Grande do Sul. Porto Alegre, Rio Grande do Sul, Brazil

**Keywords:** Occupational Stress, Nursing, Team, Occupational Health, Biofeedback, Randomized Controlled Trial, Estrés Laboral, Grupo de Enfermería, Salud Laboral, Biofeedback, Ensayo Clínico Controlado Aleatorio, Estresse Ocupacional, Equipe de Enfermagem, Saúde do Trabalhador, Biofeedback, Ensaio Clínico Controlado Aleatório

## Abstract

**Objective:**

to assess the effect of cardiovascular biofeedback on nursing staff stress when compared to an activity without self-monitoring.

**Method:**

a randomized controlled clinical trial, carried out with nursing professionals from a university hospital. The intervention group (n=58) performed cardiovascular biofeedback, and the control (n=57) performed an online puzzle without self-monitoring, totaling nine meetings over three weeks. The outcome was assessed using the Stress Symptoms and Work-Related Stress scales, and the biological marker heart rate variability. The generalized estimating equations method was used.

**Results:**

the intervention had no effect on self-reported instruments (p>0.050). However, there was an effect of time (p<0.050) on all heart rate variability indicators, demonstrating changes over the sessions.

**Conclusion:**

cardiovascular biofeedback showed promising results in the biological marker, suggesting that it can be used in nursing staff as a complementary therapy by promoting better autonomic nervous system regulation.

## RESUMO

**Objetivo:**

avaliar o efeito do *biofeedback* cardiovascular no estresse da equipe de enfermagem quando comparado a uma atividade sem automonitoramento.

**Método:**

ensaio clínico controlado aleatório, realizado com profissionais de enfermagem de um hospital universitário. O grupo intervenção (n=58) realizou *biofeedback* cardiovascular, e o controle (n=57) fez um quebra-cabeça *online* sem automonitoramento, totalizando nove encontros durante três semanas. O desfecho foi avaliado pelas escalas Sintomas de Estresse e Estresse no Trabalho, e pelo marcador biológico variabilidade da frequência cardíaca. Utilizou-se com o método Equações de Estimativas Generalizadas.

**Resultados:**

a intervenção não apresentou efeito nos instrumentos autorreferidos (p>0,050). Entretanto, houve efeito de tempo (p<0,050) em todos os indicadores da variabilidade da frequência cardíaca, demonstrando modificação ao longo das sessões.

**Conclusão:**

o *biofeedback* cardiovascular apresentou resultados promissores no marcador biológico, sugerindo que poderá ser utilizado na equipe de enfermagem como terapia complementar por promover melhor regulação do Sistema Nervoso Autônomo.

## RESUMEN

**Objetivo:**

evaluar el efecto del biofeedback cardiovascular sobre el estrés del equipo de enfermería cuando se compara con una actividad sin autocontrol.

**Método:**

ensayo clínico controlado aleatorizado, realizado con profesionales de enfermería de un hospital universitario. El grupo de intervención (n=58) realizó biorretroalimentación cardiovascular, y el control (n=57) realizó un rompecabezas en línea sin autocontrol, totalizando nueve reuniones durante tres semanas. El resultado se evaluó mediante las escalas Síntomas de Estrés y Estrés en el Trabajo, y el marcador biológico de variabilidad de la frecuencia cardiaca. Se analizó utilizando el método Generalizado de Ecuaciones de Estimación.

**Resultados:**

la intervención no tuvo efecto en los instrumentos autoinformados (p>0,050). Sin embargo, hubo un efecto del tiempo (p<0,050) en todos los indicadores de variabilidad de la frecuencia cardíaca, demostrando cambios a lo largo de las sesiones.

**Conclusión:**

el biofeedback cardiovascular mostró resultados promisorios en el marcador biológico, sugiriendo que puede ser utilizado en el equipo de enfermería como terapia complementaria al promover una mejor regulación del Sistema Nervioso Autonómico.

## INTRODUCTION

Nursing is carried out by professional categories that, although distinct, work interconnected in a wide variety of environments, and is present 24 hours a day, 365 days a year in the hospital network. Although work plays an important role in meeting basic needs and insertion in society, in nursing, there are stressors resulting from physical and mental loads, a fact that can cause illness in workers^(1)^.

Worldwide, stress is among the greatest health problems as adjunct in various physical and psycho-emotional pathologies, such as gastrointestinal problems and anxiety. Occupational stress can be defined as a process in which individuals perceive work demands as stressors, which, when exceeding their coping ability, provoke negative reactions in the subject, constituting a subjective phenomenon^(2-3)^.

Cardiovascular biofeedback (CBKF) tools, whose self-regulation and self-control processes occur through the man-machine interface, have been gaining visibility as a non-drug therapy, isolated or combined with other therapies. Investing in interventions that strengthen workers individually and in work environments can be a strategy for reducing psycho-emotional illness. Research has shown that CBKF techniques are effective in managing stress in different populations, and can help strengthen strategies for coping with adverse situations^(4-6)^.

The CBKF technique also provides heart rate variability (HRV) measurement and assessment, a biomarker that corresponds to the natural variation that occurs between heart beats or pulses, and is closely linked to hormonal response and autonomic nervous system (ANS) adaptation, triggered when the body is exposed to a stressor. Changes in HRV patterns provide a sensitive and early indicator of health impairments. A high variability in heart rate is a sign of good adaptation, characterizing a healthy individual with efficient autonomic mechanisms^(7)^.

Studies on using CBKF in nursing professionals and its benefits in this group are still scarce as well as in other categories of health workers. In 2017, a clinical trial tested CBKF on 135 nurses working in a psychiatric unit in three hospitals in Taiwan, demonstrating a significant reduction in occupational stress^(8)^. A systematic review on using CBKF for stress management cataloged 17 studies, carried out between 2000 and 2017. Research with health or nursing professionals was not found in the cited review^(4)^.

Thus, the study is justified by the need to develop skills that reduce stress, minimizing the risk of psycho-emotional illness. Therefore, the following research question emerged: what is the effect of CBKF on hospital nursing staff stress when compared to a placebo activity without self-monitoring?

## OBJECTIVE

To assess the effect of CBKF on nursing staff stress when compared to a placebo activity without self-monitoring.

## METHODS

### Ethical aspects

This research is associated with the matrix project “*Efeito do biofeedback no estresse, ansiedade e qualidade de vida profissional dos profissionais de enfermagem em um hospital universitário: ensaio clínico randomizado*”, which met ethical recommendations regarding research with human beings, obtaining approval from the Research Ethics Committee of *Hospital de Clínicas de Porto Alegre*. Currently, two articles related to this research have been published^(9-10)^. Participants confirmed their participation by signing the Informed Consent Form.

### Study design, period, and site

This is a randomized controlled clinical trial, comparing two groups, carried out from June 2020 to August 2021, at a public university hospital in southern Brazil. It was conducted in accordance with CONsolidated Standards Of Reporting Trials (CONSORT)^(11)^ recommendations and registered with Clinical Trials under 04446689.

### Population, sample, inclusion and exclusion criteria

The population consisted of nursing workers with stress symptoms, of both sexes, active in their position, admitted for more than 90 days, of any work shift, allocated in inpatient units for clinical and surgical patients, sectors chosen because they have similar characteristics in terms of infrastructure and the type of patient treated at this institution, since beds are arranged by line of care, admitting surgical patients in clinical units and vice versa. It was decided not to include sectors dedicated to hospital admission of patients with COVID-19l in order to reduce risk of bias.

Professionals on prolonged leave (social security benefit and pregnancy or lactation leave), on vacation, who had returned less than 15 days after such leave, with a pacemaker or heart rhythm pathologies, who started psychotropic medication or with cardiological alterations throughout the study, who did not complete all meetings or even those who were transferred to sectors that were not part of the survey were excluded.

Sample calculation was carried out with the support of a statistician, based on a study that showed a difference in stress levels immediately after the intervention (Cohen’s d= -0.33) as well as six weeks after the intervention (Cohen’s d= -0.68)^(12)^. Considering a one-tailed sample, significance level of 5%, power of 90%, standardized effect size (Cohen’s d) of at least 0.4 between assessments and with loss estimates of 5%, a minimum sample of 57 professionals was obtained in the intervention group (IG) and 57 professionals in the control group (CG), totaling 114 participants.

**Figure 1:**
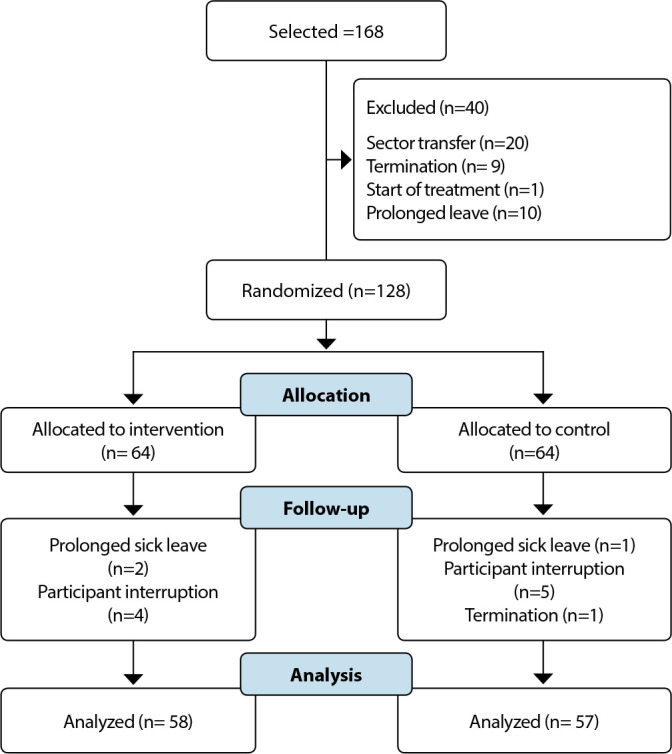
Flowchart of the participation of nursing professionals with stress symptoms in the study. Porto Alegre, Rio Grande do Sul, Brazil, 2023

Considering the aforementioned criteria, 168 nursing professionals were selected. A total of 40 were excluded before randomization; 128 were randomized; 13 were excluded after randomization, ending with 58 in IG and 57 in CG (Figure 1).

### Study protocol

To identify workers with stress symptoms in nursing staff, the researchers drew professionals from the work schedules of the sectors, using the *Sorteio de Nomes* app for Android^®^. Faced with the high absenteeism caused by the pandemic, it was decided to calculate a greater possibility of losses, and 200 participants were drawn.

The presence of stress symptoms was identified by the Stress Symptoms Scale (SSS), applied within 30 days before the initial session (t0), an instrument that provides a mean called the overall stress level (OSL)^(13)^. Professionals with OSL greater than one (OSL>1) were considered eligible and randomized in chunks, by an individual not involved in the research, through the website randomization.com, ensuring that the number of participants was equally distributed in the groups. The inclusion of subjects in the research took place gradually from June 2020 to August 2021, until reaching the minimum sample.

The intervention consisted of training the CBKF technique, using the EmWave Pro Plus^®^ software interface and interactive games, which uses photoplethysmography technology to gather and quantify, in real time, physiological data related to the heartbeat. During the interactive game, participants practice deep breathing guided by a pacer (standardized at six breaths/minute, inspiration ratio 50/50, with a pause after inspiration of 32% and after expiration of 20%, prevalent in 95% of the population), lasting 10 minutes per session. From the measured physiological behavior, the software generates continuous and dynamic information on the computer screen so that participants can gradually improve their breathing rhythm training in search of a state of self-regulation and cardiac coherence. Photoplethysmography and HRV measurement performed by the chosen software were validated in a previous research^(14)^. Check was performed on the earlobe.

The control performed an online puzzle called Jigsaw Puzzles^(15)^ on the tablet, a computerized activity without self-monitoring. A photoplethysmograph was installed in the earlobe during the game as a placebo, without measurement, aiming to maintain blinding between groups.

Considering the peculiarities of nursing professionals’ work routine, intervention and control were carried out in nine meetings, which took place three times a week, over three weeks, individually and in places close to professionals’ work sector. In the first meeting (t0), baseline HRV measurements were performed and guidance on the dynamics of the next meetings was performed. In the eight subsequent encounters (t1 to t8), CBKF training in the IG and puzzle training in the CG were carried out.

The intervention protocol consisted of the following EmWave Pro Plus^®^ games: t1 Coherence Coach; t2 Balloon Games; t3 Garden Game; t4 Rainbow Game; t5 Healing Hands Visualizer; t6 Portal of Care; t7 Child Hearts; t8 Star Fire. The CG protocol consisted of a random puzzle, from t1 to t4, a game with 16 pieces, t5/t6, with 25 pieces, and t7/t8, with 36 pieces^(15)^.

In addition to blinding participants, blinding was also considered for data analysis. For this purpose, prior to statistical consultancy, the CG and IG databases were coded in terms of participant allocation. Due to the peculiarity of the activities in the groups, it was not possible to blind the researchers.

Data collection instruments were self-administered, being delivered to participants in a brown envelope, collected on a date defined between the researched and the researcher, submitted to double typing of data in Excel spreadsheets. Participants in both groups responded to the research protocol at two moments: pre-intervention, prior to the initial or baseline session (t0), and post-intervention, immediately after the last session (t8) of the approach.

To assess the stress outcome, three variables were considered: OSL, occupational stress and HRV. OSL was assessed using the list of physical symptoms, consisting of 13 items related to physiological reactions, and the Psychological Symptoms Scale, consisting of 18 items^(13)^. For the purposes of this research, OSL was classified by tertiles, being low if values from 1.1 to 2.4, medium, from 2.5 to 3.7, and high, from 3.8 to 5.

Occupational stress was measured using the Work-Related Stress Scale (WRSS), consisting of 23 items analyzed using a 5-point Likert-type scale, in which each item presents a stressor and a type of reaction to this stressor. The scores vary between 23 and 115 points, and validity showed good reliability, with µ =0.91. The result is obtained by averaging the sum of items, considering values from 1 to 2 to be considered low level of occupational stress, from 2.1 to 2.9 to medium occupational stress, and from 3 to 5 to high occupational stress^(3)^.

HRV was transcribed using the EmWave Pro Plus^®^ HRV Assessment module. The indicators used in the research were standard deviation of all normal RR intervals recorded in a time interval (SDNN), square root of the mean square of differences between adjacent normal RR intervals, over a time interval (rMSSD), low frequency/high frequency (LF/HF) ratio and cardiac coherence. The values of HRV indicators have individual results.

### Analysis of results, and statistics

Variable analysis was performed using descriptive statistics, with the calculation of mean and standard deviation or median and interquartile range in quantitative variables, and absolute and relative frequency in qualitative variables. To compare means, Student’s t test was used, and, for proportions, Pearson’s chi-square test or Fisher’s exact test. To test the hypothesis of homogeneity of the two groups, Student’s t test was used for independent samples for quantitative variables, and the chi-square test for homogeneity was used for categorical variables. To assess the effect of the intervention, the three effect models of the generalized estimating equations (GEE)^(16)^ test were used, with Bonferroni adjustment, considering a significance level of 5% (p<0.05).

## RESULTS

Of the 200 workers drawn, 168 professionals with stress symptoms were identified, of which 128 met the inclusion criteria. Thus, 64 were allocated to GI and 64 to CG. Figure 1 presents the survey participant flow diagram. There were 522 intervention sessions and 513 control sessions.

The sample’s mean age was 43.2±8.4 years, predominantly female (96; 83.5%), 44 (38.3%) nurses, 18 (15.6%) nursing assistants and 53 (46.1%) nursing technicians. Most (100; 86.9%) worked overtime, and 65 (56.5%) used some medication. Data characterizing the sample and professionals in the groups as well as the result of intergroup homogeneity test are shown in Table 1.

**Table 1:** Sample and participant characterization by groups. Porto Alegre, Rio Grande do Sul, Brazil, 2023

Characteristics	Sample	Intervention group	Group control	*p*
	(n=115)	(n=58)	(n=57)	
Sociodemographic				
Age (years)^*^	43.2±8.4	42.3±7.5	44.0±9.3	0.283
Female^†^	96(83.5)	45(77.6)	51(89.5)	0.860
Labor				
Professional category^†^				0.995
Nurse	44(38.3)	22(37.9)	22.0(38.6)	
Nursing assistant	18(15.7%)	9(15.5)	9(15.8)	
Nursing technician	53(46.1%)	27(46.5)	26(45.6)	
Shift^†^				0.788
Day	86(74.8)	44(75.9)	42(73.7)	
Night	29(25.2)	14(24.1)	15(26.3)	
Nursing tenure (years)^*^	17.6±7.2	16.9±6.6	18.3±7.8	0.285
Job tenure (years)^*^	10.2±7.7	10.1±6.7	10.2±8.6	0.891
With another employment relationship^†^	19(16.5)	8(13.8)	11(19.3)	0.427
Work overtime^†^	100(86.9)	52(89.7)	48(84.3)	0.640
Health-related				
Any physical health problem^†^	29(25.2)	16(27.6)	13(22.8)	0.555
Use medication^†^	65(56.5)	32(55.2)	33(57.9)	0.768
Follow-up for mental health^†^	28(24.3)	13(22.4)	15(26.3)	0.626
Smoker^†^	14(12.2)	6(10.3)	8(14.0)	0.545
Drink alcohol (at least once a week)^†^	46(40)	26(44.8)	20(35.1)	0.286
Hours of sleep in the 24 hours^*^	6.7±1.4	6.6±1.5	6.8±1.4	0.346
Overweight and obesity^†^	64(55.6)	33(56.8)	31(54.4)	0.710
Use stimulating drink (> 300 ml per day)^†^	87(75.7)	46(79.3)	41(71.9)	0.357

* Média/desvio padrão (Teste t);

† n/% (Qui-Quadrado)

The non-observance of a statistically significant difference between groups is highlighted, characterizing homogeneity in the sample.

The analysis of IG and CG means showed that there was no statistically significant difference in OSL between groups (IG 2.0±0.1; CG 2.2±0.1; p= 0.356) and between sessions (D0 2.3±0.8; D8 2.1±0.8; p=0.823). Comparison of means between groups versus sessions showed a reduction in the OSL value in IG and CG, but with no statistically significant difference (IGD0 2.2±0.8; IGD8 2.1±0.8; CGD0 2.3±0.8; CGD8 2.0±0.8; p=0.531).

As for WRSS, there was no statistically significant difference in the effect of groups (IG 1.8±0.8; CG 1.9±0.8; p=0.542) and between sessions (D0 1.9±0.7; D8 1.8±0.7; p=0.199). In the groups versus sessions effect, there was a reduction in the mean of D8 in IG and CG, when compared to D0, however without statistically significant difference (IG D0 1.9±0.7; IGD8 1.7±0.8; CGD0 1.9±0.7; CGD8 1.8±0.6; p=0.169). According to the three GHG effect models, it was verified that CBKF did not present a statistically significant result in the OSL and WRSS instruments (p>0.05).

The comparison of the means of HRV indicators in the effect of groups, according to GEE, showed a statistically significant difference in SDNN (IG 73.1±4.0; 59.3±4.0; p=0.016), LF/HF ratio (IG 7.6±0.6; CG 1.6±0.2; p<0.001) and cardiac coherence (IG 60.4±1.8; CG 34.1±0.8; p<0.001). In rMSSD, however, there was no statistically significant difference between groups (IG 65.7±5.7; CG 65.4±5.9; p=0.974).

In the effect of time (sessions), there is a statistically significant difference in SDNN (D0 53.9±2.9; D8 71.4±5.4; p=0.016), LF/HF ratio (D0 2.2±0.3; D8 3.3±0.3; p=0.008), cardiac coherence (D0 39.9±1.3; D8 46.6±1.3; p<0.001) and rMSSD D0 55.7±4.9; D8 72.8±7.7; p=0.007). Table 2 presents the description of the significance level of the comparison of HRV means in the interaction model groups versus sessions.

**Table 2:** Description of the significance level (p) in the comparison of means of heart rate variability indicators according to interaction between groups (IG and CG) and sessions (D0 to D9). Porto Alegre, Rio Grande do Sul, Brazil, 2022

	Indicators							
	SDNN		rMSSD		LF/HF		Heart coherence	
	IG	CG	IG	CG	IG	CG	IG	CG
D0	58.9±4.8	49.2±3.5	54.9±6.4	56.6±7.5	2.9±0.5	1.7±0.3	43.9±2.1	35.8±1.5
D1	74.3±6.2^*^	56.8±4.6^*^	65.7±8.6	58.1±6.5	9.2±1.2^*^	1.5±0.2	64.5±2.3	36.4±1.4
D2	70.3±4.7	54.8±4.7	61.8±5.9	58.1±6.9	8.0±0.8^*^	1.5±0.2	62.1±2.4	34.2±1.0
D3	84.8±8.3^*^	64.2±5.3^*^	82.0±12.2^*^	67.9±7.4	8.2±1.1^*^	1.7±0.3	62.3±2.6	33.7±1.2
D4	77.1±6.9^*^	68.4±7.5^*^	70.5±9.8	78.7±10.6^*^	8.5±0.8^*^	3.4±1.9	61.0±2.4	32.4±1.4^*^
D5	68.4±4.1	58.7±5.3	56.2±5.1	67.3±8.1	8.9±0.8^*^	1.1±0.1	70.2±6.3	33.6±1.2
D6	74.7±4.9	58.7±5.6	66.3±7.0	65.4±7.9	8.2±0.8^*^	1.6±0.2	60.3±2.4	34.3±1.2
D7	73.3±5.4	60.8±6.8	64.6±7.7	67.5±9.8	8.8±1.0^*^	1.3±0.1	59.5±2.4	32.7±1.1^*^
D8	79.0±7.5^*^	64.5±7.5^*^	73.4±10.9^*^	72.3±11.0	8.4±8.1^*^	1.3±0.2	59.4±2.4	33.9±1.1
*p*	0.907		0.486		<0.001^*^		<0.001^*^	

* *Mean difference is significant at the 0.05 level when comparing session mean with D0.*

## DISCUSSION

The sample was homogeneous and composed of young adults, mostly female. The Nursing Profile in Brazil survey showed that there has been a rejuvenation of nursing staff, with 49.6% in the range of 31 to 45 years and 85.1% female, a phenomenon that has occurred for many decades^(17)^. It should be noted that being female is associated with higher levels of stress, probably due to the fact that women work double or triple shifts, when considering domestic work^(18-19)^.

This study identified a high number of professionals working overtime in an institution that uses overtime, which characterizes a strategy for supplementing the income of these workers. Nursing professionals’ low remuneration has been a subject discussed for years as well as the hegemony of other categories that work with shorter hours and higher wages. Excessive hours or double shifts as well as precariousness of nursing work can lead to occupational illness^(20)^. A study carried out with 584 nursing workers found that professionals with double shifts are more vulnerable to burnout^(21)^.

As for the instruments, in OSL and WRSS, it was found that CBKF did not have a statistically significant effect. This result demonstrates that there was no change in nursing professionals’ perception about the stress symptoms and occupational stress. The issue of the possibility of perpetuation of organizational factors and work-related stressors throughout the tested intervention is considered, which made it impossible for workers to perceive improvement. Also, intervention time may have been insufficient to obtain a response perception in the IG.

The OSL score that suffered the greatest decrease was in the CG, demonstrating that this mean reduction was not related to the intervention. This group may have been more sensitive to a placebo effect, as participants were blinded. A similar result occurred in a study that assessed the effect of massage and aromatherapy for stress reduction^(22)^.

On the other hand, this randomized clinical trial (RCT) aimed to assess whether using an activity with monitoring would produce a different result in terms of stress when compared to another without monitoring, which did not occur. Currently, it is known that using games, such as puzzles, can bring numerous benefits to users. A study developed with chronically stressed patients demonstrated that using games generates fun, engagement and mentally removes the sources of stress^(23)^. In this regard, the CG of this research developed the role of a second intervention.

Another clinical trial was carried out with nurses from inpatient units at the Massachusetts Hospital, which tested a program with breathing exercises for two weeks, finding no effect on stress. The authors did not raise a hypothesis for this finding, but they relate the result to the fact that nursing prioritizes care for the other to the detriment of their own, a fact that needs to be worked on in teams by organizations^(24)^.

It should be noted that this RCT was carried out during the pandemic period and that all professionals surveyed were in similar work scenarios. Moreover, other stress-generating sources occurred during collection, which are part of the institution organization, such as election for change of direction, service and unit heads as well as organizational changes for patient safety purposes and preparation for the international accreditation process.

Although there were no changes in the scales, a series of HRV indicators underwent changes throughout the research. This fact demonstrates that, even if workers did not perceive the reduction of stress symptoms or occupational stress, they were somehow present.

All HRV indicators showed a time effect, demonstrating that one or both groups changed over the sessions (p<0.050). With the exception of HF-LF ratio and cardiac coherence, the CG showed similar behavior to the IG in the other indicators, with an increase in the means in SDNN and rMSSD. This result may indicate that moving away from the work routine and concentrating on another activity, even a simple one, such as a puzzle, and for a short period (approximately 10 minutes), may bring about changes in biomarkers over time and, consequently, health benefits.

Another factor to be considered is that the professionals felt “taken care of” by someone in a moment of overload. The placebo effect involves cognitive and emotional factors, in addition to genetics and learning mechanisms, and can be added to active treatment and optimize the expected results when individuals believe that a treatment will help them^(25)^.

SDNN showed a group effect, in addition to time effect, demonstrating a difference between those who received the intervention and those who did not. The significant increase in the means of IG sessions, when compared to the means of the CG, demonstrated the effect of HRV CBKF on this indicator, which represents a summary of all HRV indicators, being considered an indicator of globality.

On the other hand, the interaction between sessions versus group did not show a significant result in IG and CG in relation to SDNN, demonstrating that the intervention time was not responsible for the increase in the means found, data that corroborates the investigation that showed a positive result in only one CBKF session^(26)^.

Increased SDNN means greater HRV, characterizing better autonomic regulation through the parasympathetic nervous system’s (PNS) prompt action when there is sympathetic activation, good physiological adaptation and, consequently, positive response in the presence of stressful situations^(27)^. An investigation assessed the effect of a single HRV CBKF training session in adults, performed through a cell phone application, finding results similar to those of this study, demonstrating that only one session of this technique can be effective in reducing the response of stress symptoms from the sympathetic nervous system (SNS)^(26)^.

The rMSSD showed only a time effect, with an increase in means in both groups, predominantly in the CG. This indicator represents an increase in vagus activity over the heart, which can be understood as a parasympathetic response to activities performed. Increased parasympathetic activity is related to times of relaxation, rest, rest, and therefore, a reduced stress response. In this sense, it can be said that, based on the result found in the CG, the puzzle activity triggered greater relaxation than CBKF. For some authors, the increase in rMSSD means adjustments in the coping response and better response to moments of adversity^(28)^.

The LF/HF ratio showed a statistically significant difference in the three GEE effect models, demonstrating that the intervention produced autonomic balance in the IG, indicating a balance between SNS and PNS, while the same did not occur in the CG. The effect of the group versus time interaction reinforces that, over time, there was a statistically significant difference in the LF/HF ratio in the comparison between those who used HRV CBKF and those who received the placebo activity. A study that compared the effect of HRV CBKF with muscle relaxation in university students identified a similar result in the LH/HF ratio, with an increase in the means in BKF HRV and maintenance of values in the muscle relaxation group^(29)^.

Using HRV CBKF protocols has shown effectiveness in different populations and, for this reason, has been used in stress management programs. Using a well-defined protocol, with slow and deep breathing training (cycles around 6 breaths per minute), increases HRV and tends to significantly increase SDNN and LF, a result found in this study^(30-31)^.

Heart coherence means showed a statistically significant difference in the three GEE models, demonstrating that HRV CBKF had an effect on this indicator. Furthermore, an increase in the mean was observed in the IG from the second session, and the same was maintained throughout the entire intervention protocol. HRV CBKF training aims to regulate physiological parameters to achieve cardiac coherence. Frequent training with well-defined protocols favors the maintenance of a state of coherence and, consequently, reduces sympathetic hyperactivation in the face of chronic stress^(32)^.

In short, in this study, the HRV CBKF intervention had an effect on indicators that represent ANS regulation (SDNN, LF/HF ratio and cardiac coherence), differing from placebo, reinforcing other similar findings identified in the literature^(33)^. rMSSD presented modification in the CG, demonstrating that the placebo activity only caused relaxation. Thus, deep breathing exercises with prolonged exhalation increase parasympathetic activation, triggering the vagus nerve, in order to promote calm and regulation of emotions^(34-35)^.

### Study limitations

This study presented as a limitation the concomitance with the pandemic, due to the number of professionals excluded due to illness, transfers and interruption of research protocol as well as increased data collection time and intervention application in research participants. Research procedures needed to be adapted to institutional protocols to prevent the spread of SARS-CoV-2, without damaging the proposed methodology. There was also a shortage of clinical trials on using HRV CBKF in nursing professionals, limiting the discussion of findings with the literature.

### Contributions to nursing, health, or public policies

On the other hand, this RCT represents advances for workers’ health and for nursing, since this study proposed a more robust methodology, aiming to verify the effect of an intervention on an outcome. Using HRV CBKF brought two strengths: encouraging self-regulation, involving workers in seeking an expected result through goals for prevention and health maintenance, and providing the measurement of a phenomenon in a non-invasive way. The results found brought a great contribution to nursing by demonstrating that HRV CBKF can improve autonomic regulation, minimizing the symptoms of chronic stress.

## CONCLUSIONS

This research made it possible to measure the effect of HRV CBKF on stress, occupational stress and resilience at work in nursing professionals. It was identified that this technique promoted changes in biological markers that provided better ANS regulation, but not in workers’ perception when responding to the stress instruments. It is suggested that these instruments be assessed in more detail at another time, seeking to verify whether there was a significant change in indicators after the intervention.

HRV assessment is a non-invasive technique that allows identifying physiological conditions in the face of stress, and CBKF is a tool that allows monitoring these changes in real time, interactively.

This study aimed to indicate that non-pharmacological measures can be used to improve nursing workers’ health conditions, professionals who are continually exposed to stress due to the content of their work. It is suggested that this investigation be expanded in the future, increasing the number of assessed professionals and different areas of activity.
